# The Relationships between Food Literacy, Health Promotion Literacy and Healthy Eating Habits among Young Adults in South Korea

**DOI:** 10.3390/foods11162467

**Published:** 2022-08-16

**Authors:** Yoojin Lee, Taehee Kim, Hyosun Jung

**Affiliations:** 1 Smart Education Platform, KyungHee University, Seoul 02447, Korea; 2 Center for Converging Humanities, KyungHee University, Seoul 02447, Korea

**Keywords:** food literacy, health promotion literacy, healthy eating habits, young adults

## Abstract

The obesity problem has reached a critical level and is threatening not only personal health but also public health systems around the world. Obesity in young adults is especially rapidly growing and many studies have confirmed that the best prevention is developing healthy eating habits with the improvement of food and health promotion literacy competencies. In this context, this study diagnoses the present levels of food literacy and health promotion literacy among young adults and explores the relationships between both literacies and their healthy eating habits. A total of 325 young adults in South Korea participated in this research, and the results are as follows. First, all food literacy components, which are food and nutrition knowledge, food skills, and resilience, are positively associated with healthy eating habits. Second, health promotion literacy is also positively associated with young adults’ healthy eating habits. Lastly, unlike the primary information sources, gender has a moderating effect on the relationships between both literacies and healthy eating habits. This indicates that the government and educational sectors should propose more policy supports and solid education systems in order to help young adults develop their food and health promotion literacies for overall well-being in the future.

## 1. Introduction

In recent years, the number of people with obesity has increased rapidly worldwide due to eating habits becoming highly westernized and irregular lifestyles being intensified as a result of global pandemics [1,2,3]. Obesity, which is caused by the energy imbalance stemming from people’s energy expenditure being low when compared with the amount of nutrients they ingest from food, was not considered a significant social problem until a few decades ago; rather, it was viewed as a symbol of wealth [4]. However, the serious side effects of obesity, such as diabetes, high blood pressure, and hyperlipidemia, are now abundantly clear, which has prompted the World Health Organization to identify obesity as a new infectious and potentially fatal disease in the 21st century [5,6]. Experts have suggested various social and environmental phenomena, including a widespread decline in home cooking and an increase in excessive drinking and smoking, to be the main causes of the surging global obesity rate [7,8].

Against this backdrop, governments around the world have started to urge members of the public to consider the perils of unbalanced lifestyles as well as the significance of healthy eating habits [9,10]. Tackling obesity is especially important because not only can obesity cause various potentially fatal diseases, it can also induce psychological problems that increase the likelihood of depression and social isolation [11,12,13]. In light of all this, governments and educational sectors are currently focusing on methods to reduce the high rate of health risks of obesity for public health, and food literacy and health promotion literacy have been investigated as possible solutions to the health problems that humans face in today’s modern society [14,15,16]. Food literacy is commonly defined as “the ability to develop into a positive interrelationship with food as well as individual food skills and behaviors within a complex food system” [9,17], while health promotion literacy is described as “the ability to access, understand and apply health information into real life in order to promote one’s good health” [18]. A number of recent studies have found that people’s daily eating habits are closely connected to their food and health promotion literacy [18,19,20], and these two forms of literacies have been suggested to empower individuals, households, communities, and even nations to enhance the quality of people’s diet and support their resilience over time [9]. Therefore, it is vital that people expand their understanding of the benefits of good food and healthy eating practices by increasing their competencies with regard to food literacy and health promotion literacy [1,17].

According to prior studies, having sufficient food literacy and health promotion literacy is particularly significant for young adults [21]. As young adulthood represents a transition period characterized by growth and changes in relation to many social, environmental, and psychological aspects [22,23], developing accurate and constructive thought with regard to healthy food and appropriate eating habits is vital [24,25]. However, the younger generations nowadays tend to prefer fast food due to its convenience and low price [26,27], and they often adopt sedentary lifestyles that contribute to weight gain due to a lack of exercise [28]. In addition, young people’s excessive exposure to social media platforms featuring provocative food commercials also encourages excessive food consumption, which may nudge people toward adopting poor eating habits as part of their daily routines [29,30,31]. It is essential to focus on engaging their interest in the improvement of both their food literacy and their health promotion literacy because the greater the degree to which young people become proficient in these two literacies, the more capable they will become of resisting the temptation of empty calorie foods, following healthy diets, and achieving balanced lives [9,25].

However, there is a dearth of quantitative research concerning food literacy and health promotion literacy that focuses on young adults. In particular, in-depth research on the relationship between young adults’ food and health promotion literacies and their eating habits is currently lacking. Furthermore, most studies regarding food literacy and health promotion literacy conducted in South Korea have primarily focused on exploring the conceptual framework of research variables or establishing valid definitions of those variables by means of qualitative approaches. To address these gaps in the literature, the present quantitative empirical study investigates the current levels of food literacy and health promotion literacy among young Korean adults by examining their food and nutrition knowledge, food skills, resilience, and health promotion competence in detail. It also examines the relationships between their food literacy, health promotion literacy and their healthy eating habits in depth. In addition, this study analyzes the moderating effects of both gender and the primary information source in an effort to uncover meaningful information with profound implications. Thus, the present study contributes to the understanding of the levels of food literacy and health promotion literacy among young adults and provides evidence of the need for effective policy supports involving systematic education plans for promoting healthy eating habits in this population. The primary aims of this study can be summarized as follows:

(1) To examine the current level of food literacy and health promotion literacy among young Korean adults.
(2) To analyze the relationships between young adults’ food literacy, health promotion literacy and their healthy eating habits in detail.
(3) To analyze the moderating effects of gender and the primary information source on the relationships among all the variables of interest.

## 2. Theoretical Background and Research Hypothesis

### 2.1. Food Literacy

As modern societies, food environments, and food systems grow more complex, the relationships among them and the individual become further complicated [32,33,34]. In this context, the concept of “food literacy” was introduced by nutritionists and food experts to explicate the diverse functions and prominent roles that food plays in people’s lives [9]. As the scope and dimensions of food literacy are both broad and multilayered, it can be interpreted in diverse ways depending on the particular researcher’s focus and the specific context of the study [1,14,35]. For instance, Vidgen and Gallegos [17] defined food literacy as “a set of planning, management, selection, preparation and daily practicalities associated with navigating the food system and using it to ensure regular food intake consistent with nutrition recommendations”, while Rawl et al. [36] described it as “the degree to which individuals have the capacity to acquire, interpret, and understand food and nutritional information needed to promote health.” Poelman et al. [16] and Cullen et al. [37] focused particularly on the social and environmental aspects of food literacy, and they define the concept as “the ability to develop into a positive relationship with food within a complex food system and to make sustainable and conscious decisions on food choice.”

As the definition and scope of the concept of food literacy vary according to the research context, there are a variety of methods available for measuring it [14,38]. One such method is the short food literacy questionnaire (SFLQ) developed by Krause et al. [15], which measures people’s practical food skills and knowledge in a clear and concise way. Another method, the self-perceived food literacy scale (SPFL) developed by Poelman et al. [16], explores the diverse dimensions of food literacy in adulthood but is also acknowledged as an efficient measuring tool for use with younger samples [25]. Na and Cho [39] developed a food literacy measurement scale that is especially customized for young Korean adults. Aside from these examples, there are numerous food literacy scales in use worldwide, all of which offer advantages in relation to particular research aims and contexts [40,41,42]. In this study, the scales developed by Poelman et al. [16] and Na and Cho [39] are used as the principal measurement instruments due to their inclusiveness and applicability to the specific research context.

### 2.2. Health Promotion Literacy

As people’s health is inextricably bound up with the foods they consume, people should pay close attention to both their food literacy and health promotion literacy together [18,39,43]. Health literacy, which represents the starting point for health promotion literacy, tends to focus on utilizing health-related information in a personal context [44,45]. It operates on the basis that health-related competencies allow people to enhance their knowledge, positive attitudes, and behaviors with regard to healthier eating and well-being [46,47]. Nutbeam analyzed the concept of health literacy from a more generic perspective in terms of the influence of declarative nutritional facts on individuals’ functional autonomy when it comes to their overall health and well-being [44,48]. As the concept of health literacy has evolved over time, the related concept of health promotion literacy has emerged [18] and is now interpreted as including a comprehensive range of skills, including the application of cognitive, practical, and socio-cultural skills [19,49].

An individual’s health promotion literacy is strongly related to the level of thought they dedicate to the prevention of chronic disease because the two issues are strongly connected to each other [50]. Therefore, many researchers have claimed that the notion of health promotion should be emphasized to provide meaningful insights into people’s overall well-being in a prevention context [19]. To reify the concept of health promotion, various subscales have been developed, which are now widely used to measure people’s ability to access, understand, and process information concerning health promotion [18]. According to the findings of prior studies, an individual’s level of health promotion literacy varies according to their age, gender, education level, and family background [51,52,53]. Generally, younger people with high socioeconomic status exhibit higher levels of health promotion literacy when compared to other groups. In addition, a positive association has been observed between food literacy and health promotion literacy, and these two forms of literacies are recognized as significant factors influencing individuals’ body mass index and chronic disease status [18]. Accordingly, an individual’s level of health promotion literacy is heavily associated with many factors and varies remarkably depending on their circumstances [54,55]. In this study, the subscales of the European Health Literacy Survey Questionnaire [18] are applied as the principal measurement instruments due to their inclusiveness and applicability to this research context.

### 2.3. Healthy Eating Habits

Food is an essential promoter of individuals’ health during the process of physical development and mental growth [9,56]. As food influences an individual’s daily quality of life and has the potential to change their overall life trajectory, making careful food choices and practicing healthy eating habits are vital [57,58,59,60]. Moreover, food serves as “the expression of values, cultures, social relations and people’s self-determination” [61], which means that people have the chance to reaffirm their cultural identity, human dignity, and control over their life course through their intimate relationship with food [62,63]. To explain the complex systemic construct of people’s experiences with food and eating [64], Furst et al. [65] developed the food choice process model, which elucidates how food and eating habits are heavily influenced by an individual’s personal code of beliefs, literacy competencies, and socio-economic factors [66,67].

It is self-evident that food plays a crucial role in individuals’ everyday life [9] and that healthy eating habits are particularly important in relation to improving their overall quality of life [68]. Healthy eating habits, defined as “eating a variety of foods that give people the important nutrients and energy” and “having regular meals with the right amount of food for maintaining one’s good health” [68], are generally evaluated through several measurement standards such as the level of balanced diet, portion control, avoiding processed foods and keeping regular eating [27]. Although many previous studies strongly emphasized that balance, quality, and timing of eating habits determine one’s well-being and entire path in life, the daily eating habits of people nowadays, especially the younger generations, are deteriorating considerably due to various environmental and practical reasons [69,70,71]. First, as contemporary food industries and technologies have developed rapidly [72], people are beginning to prefer a wide range of processed food due to the affordable price and ready availability [27,73]. Moreover, as a result of the time-pressured social environment, people are becoming increasingly dependent on eating out and snacking, which involve a high calorie intake but only limited nutrition [74,75,76]. Irregular lifestyles characterized by insufficient sleep, excessive drinking, and/or high stress levels also disrupt the secretion of the hormones that control appetite and instigate compulsive overeating [77]. Similar to many other countries, the poor eating habits of young Korean adults have been identified as a serious contemporary social problem [39,78]. If this worsening trend with regard to rising obesity levels and unhealthy eating habits continues, it will lead to significant and potentially fatal health problems for individuals as well as serious social problems for the nation as a whole [79,80]. Thus, young people need to recognize the significance of voluntarily improving their own eating habits [22], while policymakers at the national level need to implement effective measures to support the adoption of healthy eating habits and healthy lifestyles among the general public [81].

### 2.4. Relationships among the Constructs

Prior studies have found food literacy and health promotion literacy to be positively related to healthy eating habits and negatively related to the consumption of unhealthy foods [9,16]. This indicates that if people develop ideal levels of these capabilities, such as food knowledge, skills, resilience, and health consciousness, they can make better food-related choices and take care of their own health more effectively [18,19]. Furthermore, these two forms of literacies not only induce individuals to consistently practice healthy eating habits, but also positively influence others by making clear the benefits of sustaining healthy eating practices [9,82]. Accordingly, food literacy and health promotion literacy are expected to enhance individuals’ understanding of the importance of healthy eating habits in their lives [18].

#### 2.4.1. Relationship between Food and Nutrition Knowledge and Healthy Eating Habits

Knowledge regarding food and nutrition is perceived as a powerful asset that can help individuals to make wise food choices [16,39]. More specifically, if people are well informed about various food-related matters, such as essential nutrient information, food origin and seasonality, and food resource accounting and budgeting, there is a higher likelihood that they will show an interest in healthy eating habits involving a wide range of food [25]. According to Vidgen [17], food and nutrition knowledge represents a foundational component of food literacy, and these declarative aspects precipitate individuals’ healthy eating practices by awakening an interest in a healthy food environment. Moreover, Perry et al. [14] and Colatruglio and Slater [69] emphasized how a high level of food knowledge allows individuals to make intelligent decisions regarding their food choices and reduce their food expenses through robust budgeting and planning. In addition, Reynolds et al. [83] found that people who exhibit a good level of analytical ability when it comes to food labels and nutrition information make rational decisions in terms of their food choices and prefer to purchase nutritious foods. Lee, Kim, and Jung [25] noted the positive interrelatedness between students’ food knowledge and sustainable eating habits. Therefore, based on the findings of prior studies, the present study hypothesizes that young adults’ food and nutrition knowledge are positively associated with their healthy eating habits.

**Hypothesis** **1** **(H1).** *Food and nutrition knowledgeare positivelyassociated with young adults’ healthy eating habits*.

#### 2.4.2. Relationship between Food Skills and Healthy Eating Habits

Food skills such as the ability to prepare fresh ingredients, appraise food quality, and follow recipes represent key components of the practical aspects of food literacy [16]. According to Murray et al. [22] and Thomas et al. [42], an individual’s level of skill in relation to cooking and their consumption of healthy food are inextricably linked. Indeed, the higher an individual’s level of cooking skill, the more likely they recognize the importance of healthy eating habits. Na and Cho [39] asserted that practical food skills should be emphasized as a primary element of young adults’ food literacy due to promoting favorable attitudes toward healthy eating and dietary habits. In other words, both declarative knowledge of food literacy and pragmatic food skills greatly influence individuals’ ability to make balanced food choices [9]. A lack of competence with regard to food skills can serve as a major impediment to an individual’s healthy eating habits and, therefore, result in negative health outcomes throughout their life [84,85]. Based on the above, this study hypothesizes that young adults’ food skills are positively associated with their healthy eating habits.

**Hypothesis** **2** **(H2).** *Food skillsarepositivelyassociated withyoung adults’ healthy eating habits*.

#### 2.4.3. Relationship between Resilience and Healthy Eating Habits

Resilience, a psychological factor associated with food literacy, is another significant element that individuals should pay attention to [16,25]. According to recent studies, people have become more prone to experiencing anxiety and depression due to living in a fast-paced society [86], while psychological factors often have a detrimental effect on individuals’ daily eating habits [87]. Thomas et al. [42] suggested that when people experience emotional instability, it is important to build a high level of self-efficacy and resilience in order to control unhealthy food cravings. In addition, many previous studies have confirmed that young adults who exhibit dietary resilience and higher levels of self-regulation are good at avoiding overeating and sustaining healthy eating practices even when experiencing serious adverse conditions [16,25,39]. Thus, resilience should be viewed as a major aspect of food literacy. In light of this, the present study hypothesizes that young adults’ resilience is positively associated with their healthy eating habits.

**Hypothesis** **3** **(H3).** *Resilienceispositivelyassociated withyoung adults’ healthy eating habits*.

#### 2.4.4. Relationship between Health Promotion Literacy and Food Habits

Similar to food literacy, health promotion literacy is closely related to people’s eating behaviors [18]. In fact, many food literacy studies have also addressed the concepts of health, well-being, and sustainable lifestyles [19,20,21]. According to the Sponslee et al. [18], food literacy and health promotion literacy are closely linked, with high levels of both forms of literacies leading people to keep their weight down by practicing healthy eating. Lytton [46] and Rowlands et al. [47] found that people who exhibit a high level of health literacy are competent when it comes to maintaining healthy eating habits even in poor food environments. In addition, Krause et al. [15] noted that people with an unstable socioeconomic position generally exhibit low levels of both food literacy and health promotion literacy due to a lack of relevant education, which has an adverse impact on their overall well-being [88]. On the basis of prior findings, this study hypothesizes that young adults’ health promotion literacy is positively associated with their healthy eating habits.

**Hypothesis** **4** **(H4).** *Health promotion literacyispositivelyassociated withyoung adults’ healthy eating habits*.

#### 2.4.5. Moderating Role of Gender

In many food- and health-related studies, gender has been viewed as a controversial topic [9]. Males and females have different views on the significance of food and healthy lifestyles [89], with females generally exhibiting more positive attitudes toward food and good eating habits [90]. Researchers have speculated that these kinds of gender differences might be conditioned by socio-cultural factors, which have assigned females a key role in preparing and cooking food since the ancient times [91]. Therefore, many people believe that women are “better with food” than men due to their diverse experiences of handling foods [9]. The findings of Krause et al. [15] and Sponslee et al. [18] confirmed that women exhibit higher food literacy and health literacy levels than men, and they are more inclined to practice healthy eating habits in their routinely lives. Therefore, this study hypothesizes that gender has a moderating effect on the relationships between food literacy, health promotion literacy and healthy eating habits.

**Hypothesis** **5** **(H5).** *Genderhas a moderating effect on the relationships betweenyoung adults’ food literacy, health promotion literacy andtheir healthy eating habits*.

#### 2.4.6. Moderating Role of the Primary Food Information Source

As the Internet has rapidly become the most common source of information worldwide [92], people are increasingly relying on it as their main source of news regardless of the reliability (or otherwise) of the information provided [93]. This tendency to rely on certain information sources has actually never been stronger, as social media sites have rapidly pervaded practically all aspects of daily life [94]. According to previous studies, the younger generations are more willing to rely on the Internet and are more easily affected by its marketing messages, which have more direct impacts on their food purchasing decisions when compared with those of older people [30]. However, many experts have asserted that excessive dependence on media information is dangerous due to the huge risks of knowledge distortion and uncertainty in the unregulated digital world [31]. As a consequence, some young people still cling to conventional information sources such as family, friends, and acquaintances [95] and make food choices as carefully advised by them [9]. As each information source has unique characteristics and distinctiveness, the present study hypothesizes that a moderating effect will be identified between groups separated on the basis of the type of primary information source they rely on.

**Hypothesis** **6** **(H6).** *The primary information sourceshave moderating effects on the relationships betweenyoung adults’ food literacy, health promotion literacyandtheir healthy eating habits*.

## 3. Research Methodology

### 3.1. Research Model

**Figure 1:**
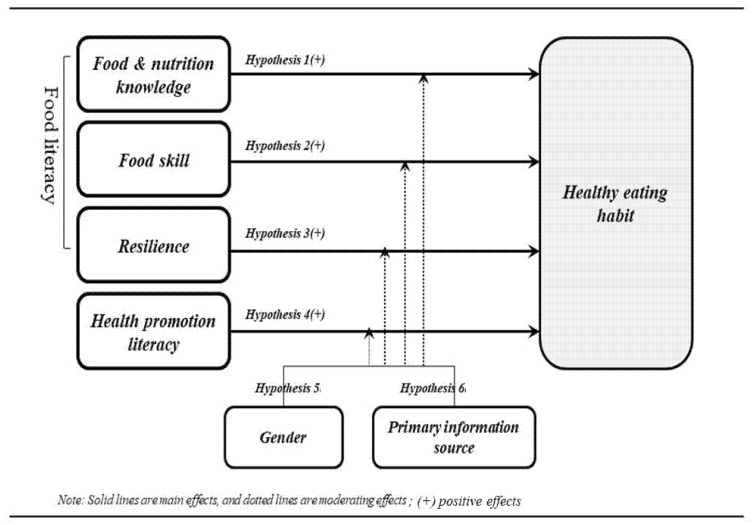
Research model.

Based on the findings of previous studies, a research model was developed for the present investigation, as shown in Figure 1. The components of the food literacy and health promotion literacy scales serve as the independent variables in the study, while the dependent variable is healthy eating habits. In addition, gender and the primary information source act as moderating variables in this study.

### 3.2. Sample and Data

The study population was defined as young adults in South Korea. A preparatory survey was conducted two weeks prior to the main survey and 100 young adults who are studying or working in universities participated in the survey. Survey questions and measurement scales were modified to fix any imprecision ambiguity based on preparatory survey results. The main research survey was executed in December 2021, when a total of 420 questionnaires were distributed through online survey system of Macromill Embrain, which is the online research agency with the largest panels in South Korea. The researchers first explained the aim of the study and promised that all the research data would be entirely processed on the condition of anonymity to the respondents, who then completed the survey using a self-administered method. A total of 365 questionnaires were returned, 325 of which were used for the final analysis after any inappropriate responses were excluded.

### 3.3. Instrument Development

A survey questionnaire in English was translated into Korean in accordance with the work of Brislin [96]. The questionnaire variables were rated on a five-point Likert scale (1 = strongly disagree; 5 = strongly agree). The operational definitions of the utilized variables are explained in Section 2 above, and all the measurement items are shown in Table 1. The questionnaire was divided into four sections. The first section measured the respondents’ food literacy levels based on the self-perceived food literacy scale developed by Poelman et al. [16] and the scale developed by Na and Cho [39]. The second section, which concerned health promotion literacy, included six items referring to the subscales of the European Health Literacy Survey Questionnaire [18]. The third section measured the respondents’ healthy eating habits based on the scales developed by Sogari et al. [27] and Lee and Lee [68]. The final section collected demographic information concerning the respondents, such as their gender, age, education level, and primary information source.

### 3.4. Data Analysis

All the statistical analyses in this study were performed using Statistical Package for the Social Sciences (SPSS) version 24.0 (IBM Corp., Armonk, NY, USA) and Amos version 24.0 software (IBM Corp., Armonk, NY, USA). A frequency analysis was employed to investigate the demographic characteristics of the study sample, while confirmatory factor and reliability analyses were performed to assess the validity and reliability of the measurement variables. The study’s hypotheses were tested using a structural equation model. Moreover, the moderating effects of gender and the primary information source were assessed using a multigroup analysis.

## 4. Results

### 4.1. Demographic Information and Measurement Model

The demographic information shows that male and female young adults accounted for 39.4% and 60.6% of the sample, respectively. In addition, young adults aged 18–24 comprised the highest proportion of respondents (85.8%) and young adults aged 25–33 made up the rest. The majority of respondents (86.2%) were enrolled in four-year university courses, with those pursuing food-related majors accounting for around 34.2% of the sample.

Table 1 summarizes the results of the validity and reliability tests of the measurement items based on the confirmative factor analysis and reliability analysis, respectively. The standardized coefficient of all the measurement items exceeded 0.6, while the t-value also showed significance (*p* < 0.001). The Cronbach’s alpha and CCR were both higher than 0.8 and the AVE exceeded 0.5, thereby satisfying the statistical significance criterion. The model’s goodness of fit was satisfactory, with χ^2^ = 757.346, *p* < 0.001, χ^2^/*df* = 2.621, NFI = 0.884, TLI = 0.915, CFI = 0.925, and RMSEA = 0.071. Table 2 presents the results of the correlation analysis of the factors, which were divided based on the results of the confirmative factor analysis. This confirms the consistency of the factors with the directions of the hypotheses in this study. Furthermore, the AVE extracted using each measurement item was higher than the square of the correlation coefficients, which confirmed the validity of the measurement items.

**Table 1:** Confirmatory factor analysis and reliability analysis.

Construct	Standardized Estimate	*t*-Value	CCR	Cronbach’s Alpha
Food and Nutrition Knowledge			0.831	0.887
FL_1_ Do you check the nutritional labels of products for calories, fat, sugar or salt content?	0.792	fixed		
FL_2_ Do you compare the calories, fat, sugar or salt content of different products?	0.748	14.347 ***		
FL_3_ If you have something to eat, do you reflect on what you have eaten earlier that day?	0.684	12.868 ***		
FL_4_ Do you purchase healthy foods, even if they are a bit more expensive? (vegetables, fruit, or whole grain products?)	0.821	16.100 ***		
FL_5_ Do you purchase healthy food, even if you have limited money? (vegetables, fruit, or whole grain products?)	0.858	17.016 ***		
Food Skills			0.892	0.921
FL_6_ Are you able to alter a recipe yourself? For example, if you are missing one of the ingredients?	0.856	fixed		
FL_7_ Are you able to prepare fresh vegetables in different ways? (cooking, steaming or stir frying, or in different dishes?)	0.836	19.020 ***		
FL_8_ Do you find it difficult to prepare a meal with more than five fresh ingredients?	0.859	19.519 ***		
FL_9_ Are you able to prepare a meal using fresh ingredients? So without pre-packed and processed foods?	0.865	20.129 ***		
FL_10_ Are you able to see, smell or feel the quality of fresh foods? (meat, fish or fruit?)	0.858	16.990 ***		
Resilience (Control Ability)			0.823	0.879
FL_11_ Are you able to say “no” to tasty snacks if you want to? (birthday treats or finger foods?)	0.781	fixed		
FL_12_ Imagine that you are at a place where you see and smell tasty foods. Are you able to resist the temptation of buying them? (at the train station, the petrol station, or at the bakery?)	0.783	14.787 ***		
FL_13_ Are you able to eat healthily when you feel stressed?	0.797	15.104 ***		
FL_14_ Are you able to control and eat the proper total contents of a bag or container of crisps, candies or cookies in one go?	0.836	16.436 ***		
Health Promotion Literacy			0.857	0.890
HPL_1_ Are you able to find various ways to relieve your stress or fatigue??	0.793	fixed		
HPL_2_ Are you able to practice helpful mental activities that promotes your well-being?	0.799	15.660 ***		
HPL_3_ Do you recognize the importance of health checkups?	0.723	13.815 ***		
HPL_4_ Do you take your family and friends’ advice on your health promotion?	0.781	15.226 ***		
HPL_5_ Do you fully understand the impact of your daily behaviors on your health?	0.680	12.824 ***		
HPL_6_ Are you able to judge the information that is helpful for preventing diseases?	0.804	16.902 ***		
Healthy Eating Habit			0.863	0.928
HEH_1_ Do you have a meal on time?	0.761	fixed		
HEH_2_ Do you have a leisurely meal without the pressure of time?	0.778	14.811 ***		
HEH_3_ Are you good at refraining from overeating?	0.790	15.064 ***		
HEH_4_ Do you try not to eat too much junk foods which are nutritionally poor?	0.898	17.602 ***		
HEH_5_ Do you try not to eat too much sweet foods?	0.871	16.964 ***		
HEH_6_ Do you try not to eat too much high-fat foods?	0.869	16.916 ***		

Note: CCR = composite construct reliability; Standardized estimate = β-value; χ^2^ = 757.346 (*df* = 289) *p* < 0.001; χ^2^/*df* = 2.621; Normed Fit Index (NFI) = 0.884; Tucker Lewis Index (TLI) = 0.915; Comparative Fit Index (CFI) = 0.925; Root Square Error of Approximation (RMSEA) = 0.071; *** *p* < 0.001.

### 4.2. Research Hypothesis

Table 3 summarizes the hypothesis test results. The final structural model exhibited a relatively excellent of goodness of fit (χ^2^ = 757.346; *df* = 289, TLI = 0.915; IFI = 0.925; CFI = 0.925; RMSEA = 0.071). In terms of the analysis results, food and nutrition knowledge were positively associated with healthy eating habits (β = 0.208; t = 2.790; *p* < 0.01), meaning that H1 was accepted. On the other hand, H2 was not accepted because the food skills variable was found to be negatively associated with healthy eating habits (β = −0.159; t = −2.754; *p* < 0.01). Contrary to food skills, both resilience (β = 0.332; t = 3.646; *p* < 0.001) and health promotion literacy (β = 0.662; t = 9.516; *p* < 0.001) were positively associated with healthy eating habits, meaning that H3 and H4 were accepted.

The present study also examined the moderating effects of differences in the respondents’ gender and primary information source on a relationship between their food literacy and health food literacy and their healthy eating habits. Prior to analyzing the moderating effects, the measurement invariance of the gender and primary information source groups was examined (Table 4 and Table 5). The difference in the chi-square value was not statistically significant at a 0.05 level, despite the difference in the degree of freedom being 10 in the invariant model, which confirmed that the metric invariance condition was satisfied. Thus, the measurement invariance of the moderating variables used in this study (gender and primary information source) was not problematic. To examine the significance of the moderating effect of gender, the difference in the degree of freedom between the constrained model and the unconstrained model was examined (Table 6). The results of the analysis showed that the relationship between resilience of food literacy and healthy eating habits was only significant among women (β = 0.346; t = 3.602; *p* < 0.001), implying that gender exerts a moderating effect in this path. Hence, only H5 was accepted. In terms of the examination of the between-group difference in significance to determine the moderating effect of the primary information source (Table 7), no difference was observed among the mass media, family, friends, and acquaintance in every path. Therefore, H6 was rejected.

## 5. Discussion and Implications

### 5.1. Conclusions and Discussion

Despite the rapid developments seen in the food industry in recent decades, young adults continue to be exposed to the significant dangers posed by unhealthy foods and poor eating habits. In light of this, the present study sought to investigate the relationships between young adults’ food literacy, health promotion literacy and their healthy eating habits. The study also examined the moderating effects of gender and the primary information source in order to extract meaningful implications.

The results of this study showed that both food literacy and health promotion literacy were closely associated with healthy eating habits. In line with the findings of prior studies, statistically significant relationships were found between food and nutrition knowledge, food skills, resilience, and young adults’ eating behaviors [9,16,39]. The most notable finding was the result that resilience is most involved with young adults’ healthy eating habits among the different dimensions of food literacy. These results indicate that young adults should be encouraged to develop not only declarative but also psychological and self-regulated aspects of food literacy in balance through a multitude of different educational and cultural ways so that they can lead healthier lives. Moreover, health promotion literacy was also significantly associated with healthy eating habits, which aligns with earlier findings [18,44,48]. This confirms that food and health are inextricably linked to each other, meaning that all the related variables need to be considered carefully in conjunction with another.

Furthermore, gender was found to play a significant moderating role between young adults’ literacies and their healthy eating habits. In particular, the female respondents scored higher in terms of their level of resilience in food literacy than the male respondents. It is assumed that since young women are generally more sensitive about their appearance than young men, they show more interest in maintaining a healthy weight and so continuously make efforts to follow balanced diet. Finally, the lack of a moderating effect on the part of the primary information source was unexpected, because the results in this study are not in line with most previous studies that examined the overwhelming influences of new information channels, such as the mass media, in comparison with traditional information channels, such as interpersonal networks. This result conclusively proved that both the latest information channels and traditional information channels play their own roles and exert significant influence on young adults’ healthy eating habits.

### 5.2. Theoretical and Practical Implications

The results of this study have several important theoretical implications. First, the study provides a starting point for considering how young adults’ food literacy and health promotion literacy capabilities are closely connected to their eating habits. Moreover, to the best of our knowledge, this is the first quantitative study to analyze the current levels of young Korean adults’ food literacy and health promotion literacy and their eating habits utilizing validated tools. Thus, it extends the existing literature by identifying the interconnectedness of all the dimensions of both food and health promotion literacies with healthy eating habits in order to inform the design of future interventions. In addition, this study addressed the concepts of food literacy and health promotion literacy from a new angle by examining the moderating effects of two types of information sources: the mass media and interpersonal networks. As the enhancement of an individual’s literacy relies heavily on both good information and a credible means of obtaining information, it is necessary to focus on the individual’s primary resource pathways when it comes to obtaining information. Although no drastic difference was identified between individuals who depend on the mass media and individuals who mostly rely on interpersonal networks, the effectiveness of each information source was clearly identified. In this regard, the present study stretched the boundaries of prior literacy studies by suggesting a new direction for future research, that is, identifying the best information tools for the enhancement of each literacy dimension.

This study also has a number of significant practical implications. First, governments, educational institutions, and researchers alike must pay more attention to the potential of both food literacy and health promotion literacy, as teaching young adults how to strengthen their literacy capabilities represents a very efficient way of targeting the burgeoning obesity problem in modern society. To achieve this, all stakeholders within the food system and the health education sector should cooperate to develop systematic intervention plans for enhancing people’s literacy competences. It would be most efficient to establish mandatory educational interventions within the school curriculum so that all individuals have the opportunity to improve their food and health promotion literacies and consistently apply it in their real lives from an early age. Such proactive educations should serve to bring about positive change in individuals’ eating behaviors, thereby helping to enhance their overall well-being in adulthood. In addition, it is noteworthy that there is no significant difference between the two groups of young adults separated on the basis of their primary information sources. This signifies that even though today’s young adults are generally stereotyped as being the digital generation, some are still influenced by their interpersonal interactions. These human communications hugely affect the way they think and the way they behave. Therefore, strategic intervention programs delivered via appropriate information channels will have synergistic effects in terms of enhancing individuals’ literacy competences. For example, specific dimensions of food literacy, such as practical food skills and psychological resilience, can be better improved through interpersonal network support, as these aspects are very difficult to develop without direct human exchanges. By contrast, people can easily expand their food- and health-related knowledge by utilizing the mass media, regardless of the location or time. Therefore, if food and health education interventions are designed carefully in light of the distinct characteristics of the different literacy dimensions and then delivered via effective information pathways, they will allow individuals to make informed decisions that promote their own health. As a consequence, the results of this study are expected to be effectively utilized to inform people about the potential influences of the two types of literacies and the importance of healthy eating by providing inspiration for the design of strategic food and health interventions in the future.

### 5.3. Limitations and Directions for Future Research

Even though this study leaves many significant considerations and future implications to researchers, it must be acknowledged that this study had a number of limitations. First, the respondents to the survey were young adults in South Korea, which means that it is difficult to generalize the results to people around the world. It is expected that additional significant results will be obtained if follow-up studies involve more diverse target groups. Moreover, as the dimensions of food literacy in this study were primarily selected to examine individual knowledge, practical skills, and behaviors, other sub-genres of food literacy (e.g., social justice and environmental aspects) were not fully addressed. As the concept of food literacy is expanding year by year, further studies with more macroscopic perspectives will also provoke meaningful discussions and generate profound implications. In addition, this study solely focused on the moderating effects of gender and the primary information source. Thus, it is necessary to verify the moderating effects of other influential factors, such as income and educational attainment. Lastly, because this study is cross-sectional study, it focused solely on the relationships between literacies and healthy eating habits, not on the causal links between them. If future studies extend the scope of the present investigation by including more research variables, their findings should provide a wealth of information that could be used to improve the public’s eating habits and encourage them to adopt healthier lifestyles.

## Figures and Tables

**Table 2:** Means, standard deviations, and correlation analyses.

Construct	1	2	3	4	5	AVE	Mean ± SD
1. Food and Nutrition Knowledge	1	*0.271* ^b^	*0.414*	*0.142*	*0.243*	*0.500*	3.52 ± 1.05
2. Food Skills	0.521 **	1	*0.302*	*0.265*	*0.045*	*0.624*	3.52 ± 1.06
3. Resilience	0.644 **	0.550 **	1	*0.116*	*0.247*	*0.538*	3.75 ± 1.07
4. Health Promotion Literacy	0.378 **	0.163 **	0.341 **	1	*0.444*	0.501	3.96 ± 0.96
5. Healthy Eating Habit	0.493 **	0.212 **	0.497 **	0.667 **	1	0.513	3.45 ± 1.28

Note: SD = Standard Deviation; ^b^ Italic type are presented in squared correlation; AVE = average variance extracted; All variables were measured on a 7-point Likert scale from 1 (strongly disagree) to 7 (strongly agree), ** *p* < 0.01.

**Table 3:** Structural parameter estimates.

	Hypothesized Path (Stated as Alternative Hypothesis)	Standardized Path Coefficients	*t*-Value	Results
H1: Food and Nutrition Knowledge → Healthy Eating Habit		0.208	2.790 **	Supported
H2: Food Skills → Healthy Eating Habit		−0.159	−2.754 **	Not Supported
H3: Resilience → Healthy Eating Habit		0.332	3.646 ***	Supported
H4: Health Promotion Literacy → Healthy Eating Habit		0.662	9.516 ***	Supported
Goodness-of-fit statistics		χ^2^_(289)_ = 757.346 (*p* < 0.001)		
		TLI = 0.915		
		IFI = 0.925		
		CFI = 0.925		
		RMSEA = 0.071		

Note: ** *p* < 0.01, *** *p* < 0.001, TLI = Tucker–Lewis Index; IFI = Incremental Fit Index; CFI = Comparative Fit Index; RMSEA = Root Mean Square Error of Approximation.

**Table 4:** Model fit indices of gender.

		χ^2^	*df*	CFI	RMSEA	RMR	∆χ^2^
Gender	Configural invariance model	1156.948	578	0.925	0.056	0.106	6.989 ^ns^
	Metric invariance model	1163.937	588	0.900	0.055	0.110	

Note: ∆*df* = 10, ∆χ^2^ = 18.307 (*p* < 0.05); CFI = Comparative Fit Index; RMSEA = Root Mean Square Error of Approximation; RMR = Root Mean Square Residual; ^ns^ Not significant.

**Table 5:** Model fit indices of primary information sources.

		χ^2^	*df*	CFI	RMSEA	RMR	∆χ^2^
Primary information sources	Configural invariance model	1184.401	578	0.925	0.057	0.116	6.055 ^ns^
	Metric invariance model	1190.456	588	0.905	0.056	0.120	

Note: ∆*df* = 10, ∆χ^2^ = 18.307 (*p* < 0.05); CFI = Comparative Fit Index; RMSEA = Root Mean Square Error of Approximation; RMR = Root Mean Square Residual; ^ns^ Not significant.

**Table 6:** Moderating effects on gender.

	Male (N = 128)		Female (N = 197)		Unconstrained Model Chi-Square (*df* = 578)	Constrained Model Chi-Square (*df* = 579)	∆χ^2^ (*df* = 1)
	Standardized Coefficients	*t*-Value	Standardized Coefficients	*t*-Value			
H5a: Food and Nutrition Knowledge → Healthy Eating Habit	0.211	1.299 ^ns^	0.232	3.064 **	1156.948	1156.966	0.019 ^ns^
H5b: Food Skills → Healthy Eating Habit	−0.123	−1.382 ^ns^	−0.149	−2.585 *	1156.948	1157.086	0.138 ^ns^
H5c: Resilience → Healthy Eating Habit	0.027	0.849 ^ns^	0.346	3.602 ***	1156.948	1163.214	6.266 *
H5d: Health Promotion Literacy → Healthy Eating Habit	0.807	6.624 ***	0.659	9.439 ***	1156.948	1159.826	2.878 ^ns^

Note: CFI = 0.925; IFI = 0.925; * *p* < 0.05, ** *p* < 0.01, *** *p* < 0.001, ^ns^ Not significant.

**Table 7:** Moderating effects on primary information sources.

	Mass Media (TV, the Internet, Social Media, Magazine, etc) (N = 255)		Family, Friends, Acquaintance (N = 70)		Unconstrained Model Chi-Square (*df* = 578)	Constrained Model Chi-Square (*df* = 579)	∆χ^2^ (*df* = 1)	
	Standardized Coefficients	*t*-Value	Standardized Coefficients	*t*-Value				
H6a: Food and Nutrition Knowledge → Healthy Eating Habit	0.181	2.158 *	0.212	2.868 *	1184.401	1185.012		0.611 ^ns^
H6b: Food Skills → Healthy Eating Habit	−0.151	−2.306 *	−0.174	−2.964 **	1184.401	1185.010		0.608 ^ns^
H6c: Resilience → Healthy Eating Habit	0.369	3.492 ***	0.349	3.893 ***	1184.401	1184.527		0.126 ^ns^
H6d: Health Promotion Literacy → Healthy Eating Habit	0.597	8.342 ***	0.640	9.200 ***	1184.401	1187.099		2.697 ^ns^

Note: CFI = 0.925; IFI = 0.925; * *p* < 0.05, ** *p* < 0.01, *** *p* < 0.001, ^ns^ Not significant.

## Data Availability

The data presented in this study are available on request from the first author.
